# Systolic blood pressure reduction with tirzepatide in patients with type 2 diabetes: insights from SURPASS clinical program

**DOI:** 10.1186/s12933-023-01797-5

**Published:** 2023-03-24

**Authors:** Ildiko Lingvay, Ofri Mosenzon, Katelyn Brown, Xuewei Cui, Ciara O’Neill, Laura Fernández Landó, Hiren Patel

**Affiliations:** 1 University of Texas Southwestern Medical Center, Dallas, TX USA; 2 Diabetes Unit, Department of Endocrinology and Metabolism, Hadassah Medical Center, Faculty of Medicine, Hebrew University of Jerusalem, Jerusalem, Israel; 3 Eli Lilly and Company, Indianapolis, IN USA

**Keywords:** Systolic blood pressure, Weight loss, Mediation analysis, Weight-loss dependent effects, Weight-loss independent effects, Tirzepatide, SURPASS studies

## Abstract

**Background:**

Tirzepatide, a once-weekly glucose-dependent insulinotropic polypeptide/ glucagon-like peptide-1 receptor agonist, is approved in the United States, Europe and Japan for the treatment of type 2 diabetes. Across the SURPASS-1 to -5 clinical studies, tirzepatide 5, 10 and 15 mg demonstrated significant improvements in glycated haemoglobin A1c (HbA1c) (− 1.9 to − 2.6%), body weight (− 6.6 to − 13.9%) and systolic blood pressure (SBP) (− 2.8 to − 12.6 mmHg) at the end of study treatment.

**Methods:**

Post-hoc mediation analyses were conducted to evaluate weight-loss dependent and weight-loss independent effects of tirzepatide on SBP reductions across the 5 SURPASS studies. The safety population (all randomized patients who took at least 1 dose of study drug) of each study was analyzed. Additional analyses were conducted at individual study level or pooled across 5 SURPASS trials.

**Results:**

The difference in mean SBP change from baseline at 40 weeks (total effect) between the tirzepatide and comparator groups was − 1.3 to − 5.1 mmHg (tirzepatide 5 mg), − 1.7 to − 6.5 mmHg (tirzepatide 10 mg) and − 3.1 to − 11.5 mmHg (tirzepatide 15 mg). These SBP reductions were primarily mediated through weight loss, with different degrees of contributions from weight-loss independent effects across the different trials. In the SURPASS-4 study, which enrolled patients with established cardiovascular disease, weight-loss independent effects explained 33% to 57% of difference in SBP change between tirzepatide and insulin glargine groups. In a pooled analysis of the SURPASS-1 to -5 studies, there was a significant (p < 0.001) but weak correlation (r = 0.18 to 0.22) between change in body weight and SBP. Reductions in SBP with tirzepatide were not dependent on concomitant antihypertensive medications at baseline as similar reductions were observed whether participants were receiving them or not (interaction p = 0.77). The largest SBP reductions were observed in the highest baseline category (> 140 mmHg), while those in the first quartile of baseline SBP category (< 122 mmHg) observed no further decrease in SBP.

**Conclusions:**

Tirzepatide-induced SBP reduction was primarily mediated through weight loss, with different degrees of contributions from weight-loss independent effects across the different trials. SBP reduction was not dependent on antihypertensive medication use but dependent on baseline SBP value, alleviating theoretical concerns of hypotension.

**Supplementary Information:**

The online version contains supplementary material available at 10.1186/s12933-023-01797-5.

## Introduction

Hypertension is a common comorbidity of type 2 diabetes (T2D) and is twice as prevalent in people with T2D compared with those without T2D [1]. Approximately half of adults with hypertension are unaware they have it and, of those with hypertension, only 42% are treated while only 21% have their hypertension under control [2]. Hypertension is a strong risk factor for microvascular and macrovascular diabetic complications, including retinopathy, nephropathy and atherosclerotic cardiovascular disease [3, 4]. The American Diabetes Association recommends that patients with T2D should achieve a blood pressure (BP) goal of less than 140/90 mmHg. For patients with a high risk of cardiovascular (CV) disease, however, they recommend a BP below 130/80 mmHg [5]. Meanwhile, the European Society of Cardiology and the European Association for the Study of Diabetes recommend a BP target of 120–130/70–80 mmHg [6].

A glucose lowering agent with clinically relevant improvements in BP and cardiovascular risk reduction may be advantageous to the majority of patients with T2D. Some glucagon-like peptide-1 receptor agonists (GLP-1 RAs) have demonstrated CV benefits, [7–10] and generally a neutral to modest reduction in BP, making them a preferred treatment option in patients with T2D with indicators of high-risk of established atherosclerotic CV disease [11]. Even small reductions of 2.4 mmHg in systolic blood pressure (SBP) can have a significant effect in reducing CV events [12], with larger SBP reductions demonstrating greater effects [13–16].

Tirzepatide, a once-weekly glucose-dependent insulinotropic polypeptide (GIP) and GLP-1 RA, is approved in the United States, Europe and Japan for the treatment of people with T2D. In five global phase 3 clinical trials (SURPASS-1, -2, -3, -4, -5), tirzepatide produced substantial reductions in glycated haemoglobin A1c (HbA1c) (− 1.9 to − 2.6%), and body weight (− 6.6 to − 13.9%) over 40 to 52 weeks, enabling many people (23–52%) with T2D to achieve normalization of glucose control (defined as HbA1c < 5.7%) [17–21]. Across the SURPASS studies, tirzepatide 5, 10 and 15 mg also demonstrated clinically relevant improvements in SBP (− 2.8 to − 12.6 mmHg) over 40–52 weeks [17–22]. As weight loss is known to lower SBP, it is important to assess the contribution of tirzepatide-induced weight loss on SBP reduction [23].

Tirzepatide has a safety profile consistent with that of GLP-1 RAs, with mild to moderate gastrointestinal adverse events (AEs) mostly reported during the dose escalation period which decreased over time. Additionally, in a meta-analyses conducted across seven phase 2 and 3 clinical studies, tirzepatide demonstrated CV safety when compared with pooled comparators with the hazard ratio of 0.80 (95% confidence interval [CI]: 0.57, 1.11) for major adverse cardiovascular events (MACE-4) which included death due to CV cause, myocardial infarction, stroke and hospitalization for unstable angina [24].

The objective of this report is to provide an overview of the effect of tirzepatide on SBP across the five SURPASS studies and to assess the impact of weight loss and other select variables (use of antihypertensive medication and baseline SBP value) on this effect.

## Materials and methods

### Study design and participants

A database was created using 40/42-week clinical data from five randomized controlled trials, SURPASS-1, -2, -3, -4 and -5. A common 40/42-week primary time point was selected for consistent assessment across the five SURPASS studies. The study design for each trial is described in detail in Additional file 1. Key eligibility criteria, and primary efficacy and safety results have been published previously for all five trials [17–21] (ClinicalTrial.gov Identifiers: NCT03954834, NCT03987919, NCT03882970, NCT03730662, NCT04039503). Two of the trials were placebo-controlled (SURPASS-1 and -5) while the remaining three trials compared tirzepatide (5 mg, 10 mg and 15 mg) to semaglutide 1 mg, titrated insulin degludec and titrated insulin glargine (SURPASS-2, -3 and -4, respectively). Participants randomized to tirzepatide started at a 2.5 mg dose once weekly and escalated the dose by 2.5 mg every 4 weeks until they reached their assigned dose. Participants continued their baseline antihypertensive medications and were permitted to adjust during the study.

Body weight measurements were carried out in a consistent manner using a calibrated electronic scale in kilograms. All weights for a given patient were measured using the same scale and patients wore light clothes with no shoes while their weight was measured. All laboratory parameters were assessed in a central laboratory. Blood pressure and pulse rate were measured after the participant sat quietly for 5 min. For each parameter, two measurements were taken using the same arm with the recordings taken at least 1 min apart. BP was taken with an automated blood pressure machine.

### Statistical analysis

Post-hoc mediation analyses were conducted to evaluate weight-loss dependent (WL-D) and weight-loss independent (WL-IND) effects of tirzepatide on SBP reductions across the five SURPASS studies individually and also pooled doses per study. The model for the estimation of WL-D and WL-IND effects on SBP at Week 40/42 included the interaction between treatment and weight change, with the baseline variable for SBP, use of antihypertensive drug, country and HbA1c category ([≤ 8.0%, > 8.0%] for SURPASS-5, [≤ 8.5%, > 8.5%] for other studies) as covariates in the model. The safety population (all randomly assigned patients who took at least one dose of study drug) of each study was used in this analysis which included data regardless of adherence to study drug or initiation, modification or discontinuation of antihypertensive medications. With the integrated database from the five studies, subgroup analyses of change from baseline in SBP by baseline antihypertensive drug use (Yes, No) was performed. Correlation between SBP change from baseline and weight change from baseline were performed and the Pearson correlation coefficient was presented. SBP change from baseline was summarized by the baseline SBP quartile.

## Results

### Baseline characteristics and key demographics

Patient demographics and baseline characteristics from participants randomized in SURPASS-1 to -5 (n = 4199; n = 1394 receiving tirzepatide 5 mg, n = 1397 receiving tirzepatide 10 mg, n = 1408 receiving tirzepatide 15 mg and n = 2064 receiving placebo or active comparators) are shown in Table 1. Blood pressure at baseline is also shown in Additional file 2. Baseline characteristics and demographics were well balanced between tirzepatide and comparators for each study and pooled dataset.

**Table 1:** Baseline characteristics and key demographics (SURPASS 1–5 individual [pooled arms] and pooled data)

	Individual SURPASS trials					Pooled SURPASS trials	
	SURPASS-1 (N = 478)	SURPASS-2 (N = 1878)	SURPASS-3 (N = 1437)	SURPASS-4 (N = 1995)	SURPASS-5 (N = 475)	Pooled TZP (N = 4199)	Pooled comparator (N = 2064)
Age (years)	54.1 ± 11.9	56.6 ± 10.4	57.4 ± 10.0	63.6 ± 8.6	60.6 ± 9.9	58.5 ± 10.4	60.3 ± 10.3
Sex—male (n, %)	247 (51.7)	882 (47.0)	802 (55.8)	`1246 (62.5)	264 (55.6)	2245 (53.5)	1196 (57.9)
Duration of diabetes (years)	4.7 ± 5.4	8.6 ± 6.5	8.4 ± 6.2	11.8 ± 7.5	13.3 ± 7.3	9.38 ± 7.0	10.12 ± 7.3
Cardiovascular disease (%)^a^	5	8	13	87	18	35	35
HbA1c (%)	7.9 ± 0.9	8.3 ± 1.0	8.2 ± 0.9	8.5 ± 0.9	8.3 ± 0.9	8.3 ± 1.0	8.3 ± 0.9
BMI (kg/m^2^)	31.9 ± 6.6	34.2 ± 6.9	33.5 ± 6.1	32.6 ± 5.5	33.4 ± 6.1	33.4 ± 6.3	33.0 ± 6.1
Weight (kg)	85.9 ± 19.8	93.7 ± 21.9	94.3 ± 20.1	90.3 ± 18.7	95.2 ± 21.6	92.6 ± 20.6	91.6 ± 20.1
eGFR (mL/min/1.73m^2^)	94.1 ± 19.7	96.0 ± 17.1	94.1 ± 17.0	81.3 ± 21.1	85.5 ± 17.8	91.0 ± 19.5	87.8 ± 20.3
SBP (mmHg)	127.6 ± 14.1	130.6 ± 13.8	131.5 ± 13.3	134.4 ± 15.4	137.9 ± 15.7	132.0 ± 14.5	133.1 ± 14.9
DBP (mmHg)	79.4 ± 8.8	79.2 ± 9.0	79.2 ± 8.9	78.4 ± 9.4	80.7 ± 10.8	79.0 ± 9.2	79.1 ± 9.4
Antihypertensive medication use (%)^b^	47	64	70	93	75	72	78
ACE inhibitors	13	30	33	40	36	32	35
Angiotensin II receptor blockers	22	26	21	37	27	27	30
Dihydropyridine derivatives	11	12	17	27	30	18	21
Beta-blocking agents	7	11	23	41	30	22	28

Data are mean ± SD, unless otherwise indicated

*ACE* angiotensin-converting enzyme *BMI* body mass index; *DBP* diastolic blood pressure; *eGFR* estimated glomerular filtration rate; *HbA1c* glycated hemoglobin A1c; *N* population size; *n* sample size; *SBP* systolic blood pressure; *SD* standard deviation; *TZP* tirzepatide

^a^Data presented for all randomised patients and for cardiovascular disease includes history of myocardial infarction, coronary revascularization, hospitalization for unstable angina or heart failure, stroke or transient ischemic attack, peripheral arterial disease, lower extremity arterial revascularization, carotid revascularization, or documented coronary artery disease

^b^Most frequently used classes of antihypertensive medications

Across the SURPASS studies, mean age was 54–64 years and 47–94% of participants were using antihypertensive medications at baseline. At baseline, SURPASS-1 participants had the lowest SBP and duration of diabetes (127.6 mmHg and 5 years) compared to SURPASS-5 participants (137.9 mmHg and 13 years). As expected, SURPASS-4 participants had the highest prevalence of CV disease (87%), use of antihypertensive medication at baseline (94%) and lowest estimated glomerular filtration rate (81.3 mL/min/1.73m^2^), as this study enrolled patients with a high CV risk (coronary heart disease, peripheral arterial disease, cerebrovascular disease, chronic kidney disease or congestive heart failure). SURPASS-4 participants were also older and 63% were male.

### Systolic blood pressure reduction with tirzepatide across SURPASS program

Across the SURPASS program, SBP reductions ranged from − 4.2 to − 12.6 mmHg in participants receiving tirzepatide. In each study, SBP reductions were greater with tirzepatide than with placebo or active comparator groups at Week 40/42.

**Fig. 1:**
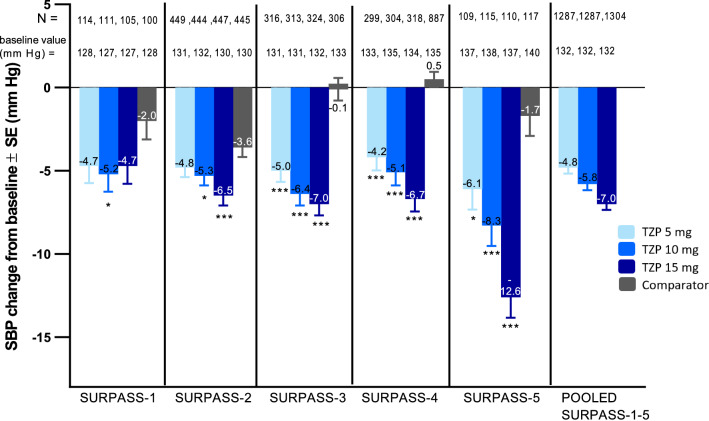
Change from baseline in systolic blood pressure at Week 40/42. Data are least-squares mean ± SE. Pooled comparator data are not presented as these comparators have varying effects on SBP. Data are taken from the safety population of each study. *p < 0.05 vs. placebo/active comparator, ***p < 0.001 vs. placebo/active comparator. *CI* confidence interval, *SBP* systolic blood pressure, *SE* standard error, *TZP* tirzepatide

In the monotherapy placebo-controlled study (SURPASS-1), treatment with tirzepatide 10 mg resulted in significantly greater SBP reductions compared with placebo (estimated treatment difference [ETD] [95% CI] − 3.1 [− 6.2, 0.1] mmHg; P = 0.04). In the add-on to basal insulin placebo-controlled study (SURPASS-5), all doses (5, 10 and 15 mg) resulted in significantly greater SBP reductions compared with placebo (ETD [95% CI] − 4.4 [− 7.8, − 1.0], − 6.6 [− 9.9, − 3.2] and − 10.9 [− 14.3, − 7.5] mmHg; P = 0.01, P < 0.001 and P < 0.001, respectively) (Fig. 1).

In the SURPASS-2 study, 10 mg and 15 mg dose groups of tirzepatide demonstrated significantly greater SBP reductions than semaglutide 1 mg (ETD [95% CI] − 1.8 [− 3.4, − 0.1] and − 3.0 [− 4.6, − 1.3] mmHg; P = 0.03 and P < 0.001, respectively), while in SURPASS-3 and SURPASS-4 studies, SBP reductions were greater with all tirzepatide doses compared with insulin degludec (ETD [95% CI] − 4.9 [− 6.8, − 3.0], − 6.3 [− 8.2, − 4.4], − 6.9 [− 8.8, − 5.0] mmHg) and insulin glargine (ETD [95% CI] − 4.7 [− 6.5, − 3.0], − 5.6 [− 7.4, − 3.9], − 7.2 [− 8.9, − 5.5] mmHg) for tirzepatide 5, 10 and 15 mg, respectively: P < 0.001) (Fig. 1).

Overall, SBP reductions were greater with tirzepatide than with placebo or active comparator groups and were dose dependent with the greatest SBP reductions observed in the tirzepatide 15 mg treatment groups. Similarly, pooled analysis across the five SURPASS trials at Week 40/42 indicated dose dependent SBP reductions of − 4.8, − 5.8 and − 7.0-mmHg for tirzepatide 5, 10 and 15 mg treatment groups, respectively. (Fig. 1).

### Association between systolic blood pressure and weight change from baseline

There were similar reductions in body weight for all tirzepatide doses across the five SURPASS studies. Similar to SBP, mean body weight reductions at Week 40/42 were dose dependent, with the greatest reductions observed in the tirzepatide 15 mg treatment groups across the SURPASS program. (− 7.0, − 9.1 and − 10.8 kg for tirzepatide 5, 10 and 15 mg treatment groups, respectively). Body weight reductions did not reach a plateau.

**Fig. 2:**
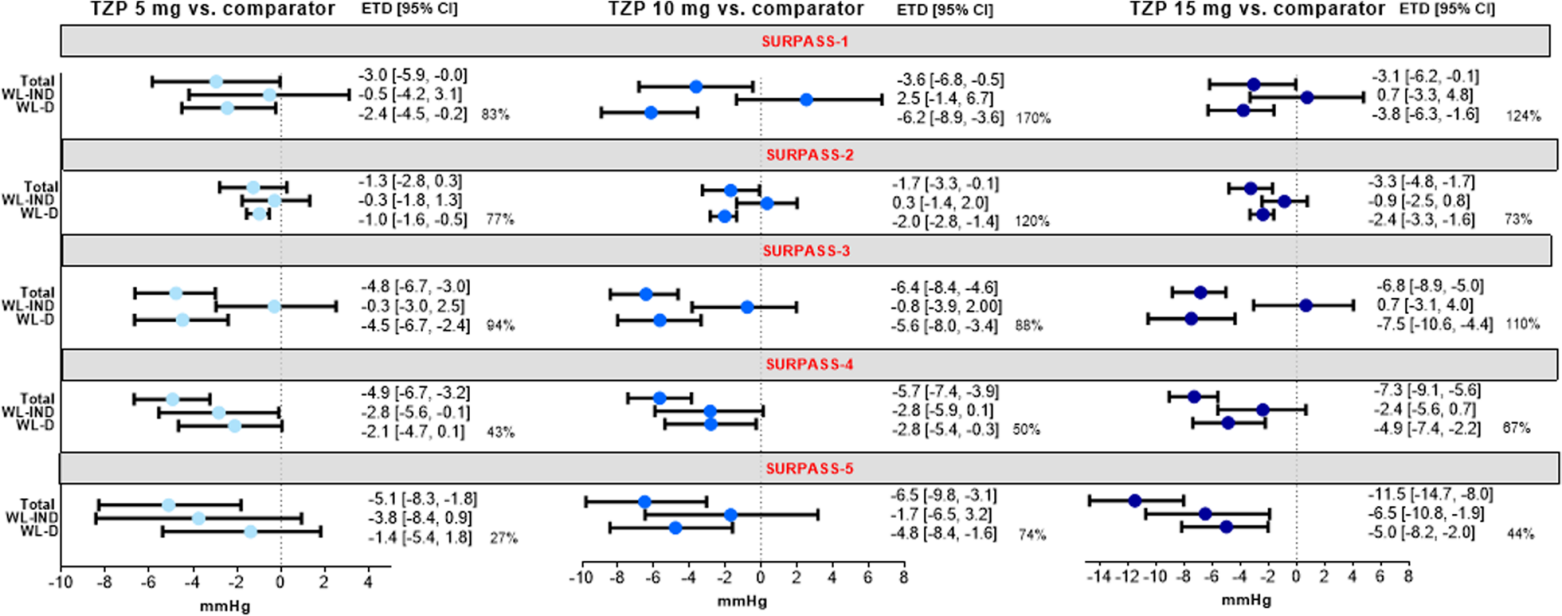
Mediation analyses for systolic blood pressure using weight loss as a factor at Week 40/42 (SURPASS 1–5 individual data). Data are least-squares mean ETD (95% CI). Data are taken from the safety population of each study. Percentage values represent the percent of blood pressure reduction mediated by weight loss. *CI* confidence interval, *ETD* estimated treatment difference, *SBP* systolic blood pressure, *TZP* tirzepatide, *WL-D* weight-loss dependent, *WL-IND* weight-loss independent

The mediation analysis showed contribution of WL-D and WL-IND effects on total effect of SBP reductions presented as difference between tirzepatide and comparator group for each study (Fig. 2). For WL-D effects between tirzepatide and comparator groups, the ETD (95% CI) in mean SBP change from baseline ranged from − 1.0 (− 1.6, − 0.5) to − 4.5 (− 6.7, − 2.4) mmHg (tirzepatide 5 mg), − 2.0 (− 2.8, − 1.4) to − 6.2 (− 8.9, − 3.6) mmHg (tirzepatide 10 mg) and − 2.4 (− 3.3, − 1.6) to − 7.5 (− 10.6, − 4.4) mmHg (tirzepatide 15 mg) (Fig. 2). WL-IND effects contributed to a lesser extent as the ETD (95% CI) in mean SBP change from baseline between tirzepatide and comparator groups ranged from − 0.3 (− 1.8, 1.3) to − 3.8 (− 8.4, 0.9) mmHg (tirzepatide 5 mg), + 2.5 (− 1.4, 6.7) to − 2.9 (− 5.9, 0.1) mmHg (tirzepatide 10 mg) and + 0.7 (− 3.3, 4.8) to − 6.5 (− 10.8, − 1.9) mmHg (tirzepatide 15 mg) (Fig. 2). Mediation analysis conducted by pooling patients across all doses of tirzepatide within each study also showed consistent results (Additional file 3).

In the SURPASS-4 study where patients with high CV risk were enrolled, and in the SURPASS-5 study where patients had the longest duration of T2D, WL-IND effects explained 33–57% and 26–73% of the difference in SBP change between tirzepatide versus the insulin glargine and placebo group, respectively (Fig. 2).

**Fig. 3:**
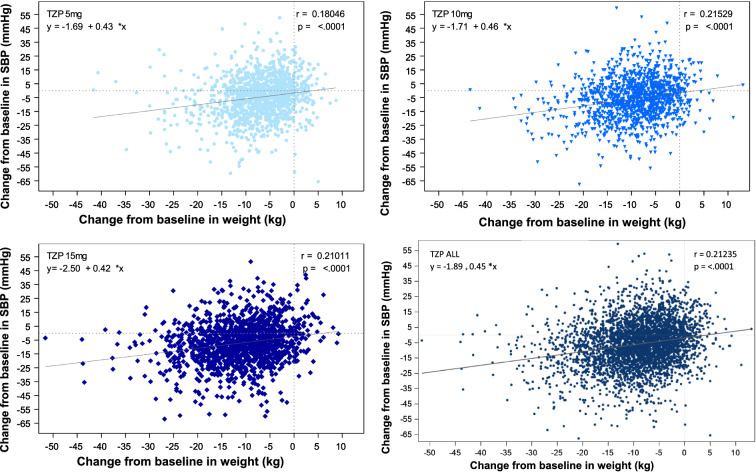
Correlation between change in systolic blood pressure and body weight at Week 40/42. Data taken from the safety population of SURPASS 1–5 pooled. *SBP *systolic blood pressure, *r* correlation coefficient, *TZP* tirzepatide

In the pooled analyses, there was a significant (p < 0.001) but weak correlation (r = 0.18 to 0.22) between change in body weight and SBP from baseline at Week 40/42 in tirzepatide-treated patients (Fig. 3).

### Subgroup analysis of systolic blood pressure change from baseline by use of antihypertensive medications

At baseline, in the pooled tirzepatide treatment groups, 72.4% of participants were receiving antihypertensive medications. Similarly, in the pooled comparator group, 78.1% were receiving antihypertensive medications at baseline. In the SURPASS-4 study which enrolled participants with established CV disease, 94% were receiving antihypertensive medications at baseline. (Table 1).

**Fig. 4:**
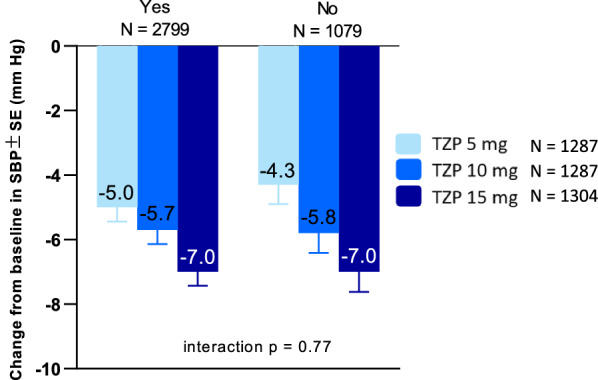
Change from baseline in systolic blood pressure at Week 40 by use of antihypertensive medication at baseline. Data taken from the safety population of SURPASS 1–5 pooled. *SBP* systolic blood pressure, *r* correlation coefficient, *TZP* tirzepatide

Reductions in SBP with tirzepatide were not dependent on concomitant antihypertensive medications as similar reductions were observed whether participants were receiving them or not (tirzepatide 5 mg, − 5.0 vs − 4.3 mmHg; tirzepatide 10 mg, − 5.7 vs − 5.8 mmHg; tirzepatide 15 mg, − 7.0 vs − 7.0 mmHg) with a non-significant treatment by antihypertensive medication (Yes, No) interaction (P = 0.77) (Fig. 4).

### Subgroup analysis of systolic blood pressure changes from baseline by quartile of baseline value

**Fig. 5:**
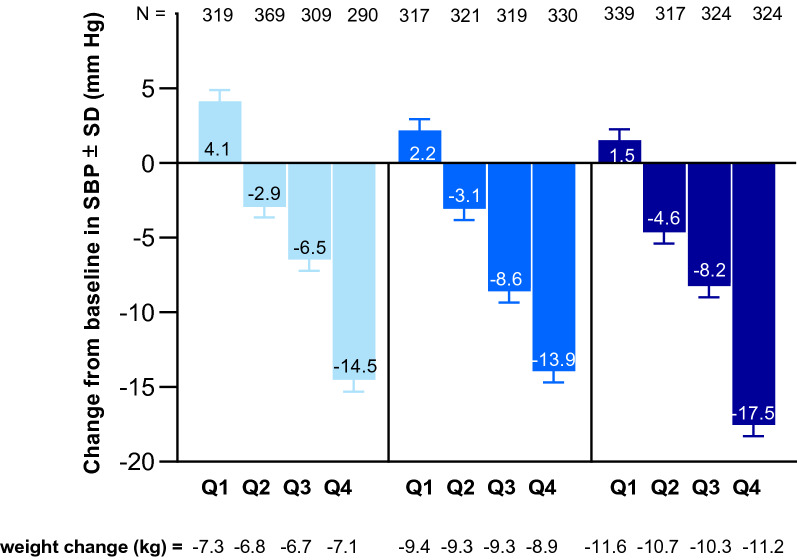
Change from baseline in systolic blood pressure at Week 40 by quartile of baseline value. Data are mean ± SE. Data taken from the safety population of SURPASS 1–5 pooled. *N* population size, *SBP* systolic blood pressure, *SD* standard deviation, *TZP* tirzepatide

The fourth quartile of SBP baseline value was > 140 mmHg, the median was 132.0 mmHg and the first quartile was ≤ 122.5 mmHg. Quartile of baseline SBP value significantly influenced the SBP change at Week 40/42 (p < 0.0001) (Fig. 5). The greatest SBP change with tirzepatide doses, which ranged from − 14.0 to − 17.5 mmHg, was observed in participants with the highest SBP values at baseline (Q4, > 140 mmHg). SBP was significantly reduced in all categories, except for in the lowest SBP baseline value category (Q1, ≤ 122.5 mmHg), where no clinically meaningful changes were observed.

Body weight reduction was similar across all the quartiles of SBP baseline value (tirzepatide 5 mg − 6.8 kg to − 7.3 kg; tirzepatide 10 mg − 8.9 kg to − 9.4 kg; tirzepatide 15 mg − 10.3 kg to − 11.6 kg) (Fig. 5).

### Safety assessment

Tirzepatide had a safety profile consistent with that of GLP-1 RAs with the majority of gastrointestinal AEs noted during the dose escalation period and decreasing overtime [17–21]. The most commonly reported gastrointestinal AEs were nausea and diarrhea. The percentage of patients reporting ≥ 1 treatment-emergent gastrointestinal AE by preferred term ranged from 3 to 16% for tirzepatide 5 mg, 3–24% for tirzepatide 10 mg and 6–24% for tirzepatide 15 mg across the SURPASS studies.

Treatment with tirzepatide resulted in a mean increase in heart rate of 1–4, 2–4 and 3–6 beats per minute (bpm) for 5-, 10- and 15-mg groups, respectively, at the end of study treatment (Week 40/42 for SURPASS-1, 2 and 5 and Week 52 for SURPASS-3 and 4). For the two placebo-controlled studies, heart rate increased by 0–2 bpm on average. Treatment with active comparators semaglutide 1 mg, insulin degludec and insulin glargine resulted in mean heart rate increases of 4, 1 and 1 bpm, respectively.

## Discussion

### Our findings in context

Tirzepatide demonstrated clinically significant improvements in SBP ranging from 4 to 13 mmHg reduction across the three doses (5 mg, 10 mg and 15 mg) at 40 weeks in the SURPASS clinical program. Tirzepatide 10 mg and 15 mg demonstrated statistically significant reductions in SBP compared to GLP-1 RA, semaglutide 1 mg (5.3 mmHg and 6.5 mmHg vs 3.6 mmHg, respectively). The magnitude of effect was also consistent in SURPASS 4 which enrolled patients with established cardiovascular disease.

We observed that the effect of tirzepatide on SBP was mediated through weight loss with different degrees of contributions from weight-loss independent effects across the different SURPASS trials. Furthermore, SBP reduction was not dependent on antihypertensive medication use but dependent on baseline SBP value.

These findings are in agreement with a pooled analysis of six randomized phase 3 clinical trials in GLP-1 RA liraglutide [25]. Similarly, the authors reported that SBP was weakly correlated with weight loss at 26 weeks and SBP reductions were observed in the presence and absence of antihypertensive medication. However, mediation analyses were not carried out across the trials to determine the extent of the weight-loss contribution, nor was the SBP change according to baseline values evaluated. In a meta-analysis conducted across 33 randomized studies, the authors evaluated GLP-1 RAs, liraglutide and exenatide, using random-effect analysis and concluded that these GLP-1 RAs induced a small but significant change in SBP that appeared to be independent of the degree of weight loss and SBP values at baseline [26].

### The effect of weight loss and other variables on blood pressure

Weight loss of about 5–10% is expected to improve SBP by > 5 mmHg [27]. Across the SURPASS program, weight loss associated with tirzepatide explained the majority of treatment effect on SBP. Excess adiposity, insulin resistance, inflammation and higher oxidative stress are hallmarks of type 2 diabetes and obesity, which are proven to alter endothelial dysfunction and affect haemodynamics resulting in elevated blood pressure [28, 29]. It is not surprising that robust weight loss, reduction in liver and abdominal fat [30] and improvement in insulin sensitivity [31] associated with tirzepatide treatment may likely affect this key pathophysiological state associated with elevated BP in this population.

Nevertheless, there was some heterogeneity in this observation in the studies with higher baseline age, SBP, use of antihypertensive medications and duration of diabetes (SURPASS-4 and SURPASS-5) where weight loss explained roughly half of the total effect on SBP. Reduction in insulin usage in the SURPASS-5 study, particularly in the 15-mg dose group, may have also played a role in SBP reduction. There was a weak correlation between weight loss and SBP in the pooled analyses across SURPASS trials which further raises curiosity to explore potential weight-loss independent mechanisms that could be driving the reduction in SBP. These mechanisms are potentially not related to the study treatment. While the mediation analysis indicated the treatment effect on SBP reduction were mainly through the weight loss, the results of the two analyses are not contradictory. The lack of robust association between weight loss and improvement in SBP has been reported with GLP-1 RAs [24, 25]. Natriuresis [32], direct vasodilation [33], reductions in sympathetic nervous system activity [34], extracellular volume and midregional-pro-atrial natriuretic peptide (proANP) [33, 35] are potential direct mechanisms for GLP-1 RAs leading to SBP lowering.

To ascertain whether use of antihypertensive mediation impacts the degree of SBP reduction with tirzepatide, analyses were conducted for subgroups of patients using or not using antihypertensive medication at treatment initiation. These findings may be relevant for health care professionals initiating tirzepatide in patients with T2D using antihypertensive medications, as this could be a potential opportunity to adjust antihypertensive medications if target blood pressure is reached. SBP reduction with tirzepatide was highest (mean of 14–18 mmHg) in patients with a baseline value greater than 140 mmHg while there was minimal impact on SBP for patients with a baseline value of less than 123 mmHg. This is a clinically relevant finding from a patient safety perspective as this effect minimizes any potential risk of hypotension or syncope. This finding is also consistent with that reported with liraglutide [36].

Although much is known about the effects of GLP-1 RAs on blood pressure, little clinical data are available on the effects of GIP agonism. After a 6-day subcutaneous GIP infusion in patients with type 1 diabetes, a 4.6 mmHg reduction in SBP was noted [37]. In a separate study on patients with T2D already using GLP-1 RA and metformin, continuous acute infusion of GIP (6 pmol/kg/min) resulted in significant reduction in SBP compared to placebo. This effect was hypothesized to be due to elevated proANP and suggests an additive haemodynamic effect of GIP and GLP-1 receptor co-agonism [38]. Superior SBP lowering with tirzepatide compared to semaglutide may be due to greater weight loss or partly due to GIP specific mechanisms that warrant further exploration.

### Safety

Across SURPASS studies, tirzepatide was associated with an increase in heart rate of 1 to 6 beats per minute at the end of study treatment. There was no significant difference between tirzepatide and semaglutide in the SURPASS-2 study at Week 40 in change in heart rate compared to baseline despite higher reduction in HbA1c, weight and SBP [18]. Several mechanisms have been postulated for elevation in heart rate with GLP-1 RAs, such as reflex tachycardia, increase in sympathetic nervous system activity and direct sino-atrial node action but none have been clinically proven yet [39]. Several long-acting GLP-1 RAs have demonstrated CV protection in a dedicated CV outcome trial (CVOT) [7–10, 38] while the SURPASS-CVOT study (NCT04255433) for tirzepatide is ongoing and will provide further insights into whether these effects on blood pressure lowering, improvements in metabolic parameters and elevation in heart rate would combine to produce any meaningful impact on hard CV outcomes. To date, tirzepatide has demonstrated CV safety when compared with pooled comparators in a meta-analysis of phase 2 and phase 3 studies with the hazard ratio of 0.80 (95% CI: 0.57–1.11) for MACE-4 [24]. In a post-hoc analysis of SURPASS-4 data, tirzepatide slowed the rate of decline in eGFR, showed clinically meaningful improvement in albuminuria and significantly lowered occurrence of the composite kidney outcomes compared to insulin glargine which may be related to SBP lowering [40].

### Limitations and conclusions

The limitations of this study include its post-hoc nature and the fact we did not systematically collect indications for antihypertensive medications as these could have been used for other co-morbidities. Studies were not designed to systematically assess blood pressure and randomization was not stratified based on baseline status of hypertension, SBP and other relevant parameters that could have affected the outcomes. Weight loss with tirzepatide did not plateau at 40/42 weeks and therefore longer term data could provide more robust assessments in future.

In conclusion, tirzepatide has demonstrated clinically relevant reductions in SBP across the SURPASS program. This effect was primarily mediated through weight loss, with different degrees of contributions from weight-loss independent effects across the different SURPASS trials. Furthermore, SBP reduction was not dependent on antihypertensive medication use and dependent on baseline SBP value, alleviating theoretical concerns of hypotension in patients with lower baseline SBP.

## Supplementary Information

**Additional file 1**: Summary of the SURPASS 1–5 study designs.

**Additional file 2**: Baseline blood pressure across SURPASS studies.

**Additional file 3**: Mediation analyses for systolic blood pressure using weight loss as a factor at Week 40/42 (SURPASS 1–5, pooled data for TZP dose).

## Data Availability

Lilly provides access to all individual participant data collected during the trial, after anonymization, with the exception of pharmacokinetic or genetic data. Data are available to request 6 months after the indication studied has been approved in the US and EU and after primary publication acceptance. No expiration date of data requests is currently set once they are made available. Access is provided after a proposal has been approved by an independent review committee identified for this purpose and after receipt of a signed data sharing agreement. Data and documents, including the study protocol, statistical analysis plan, clinical study report, blank or annotated case report forms, will be provided in a secure data sharing environment for up to 2 years per proposal. For details on submitting a request, see the instructions provided at vivli.org.

## Abbreviations

AE: Adverse event
BMI: Body mass index
BP: Blood pressure
CI: Confidence interval
CV: Cardiovascular
CVOT: Cardiovascular outcome trial
DBP: Diastolic blood pressure
ETD: Estimated treatment difference
GIP: Glucose-dependent insulinotropic polypeptide
GLP-1 RA: Glucagon-like peptide-1 receptor agonist
HbA1c: Glycated haemoglobin A1c
MACE-4: Major adverse cardiovascular events
proAND: Pro-atrial natriuretic peptide
SBP: Systolic blood pressure
T2D: Type 2 diabetes
WL-D: Weight-loss dependent
WL-IND: Weight-loss independent
