# Supplementary material for: Systolic blood pressure reduction with tirzepatide in patients with type 2 diabetes: insights from SURPASS clinical program
Source: Cardiovasc Diabetol. 2023 Mar 24;22:66. doi: 10.1186/s12933-023-01797-5 (PMC10039543; doi:10.1186/s12933-023-01797-5)
Supplement: Supplementary file 1 — Additional file 1: Summary of the SURPASS 1–5 study designs. [file 12933_2023_1797_MOESM1_ESM.docx]

Additional file 1. Summary of the SURPASS 1-5 study designs
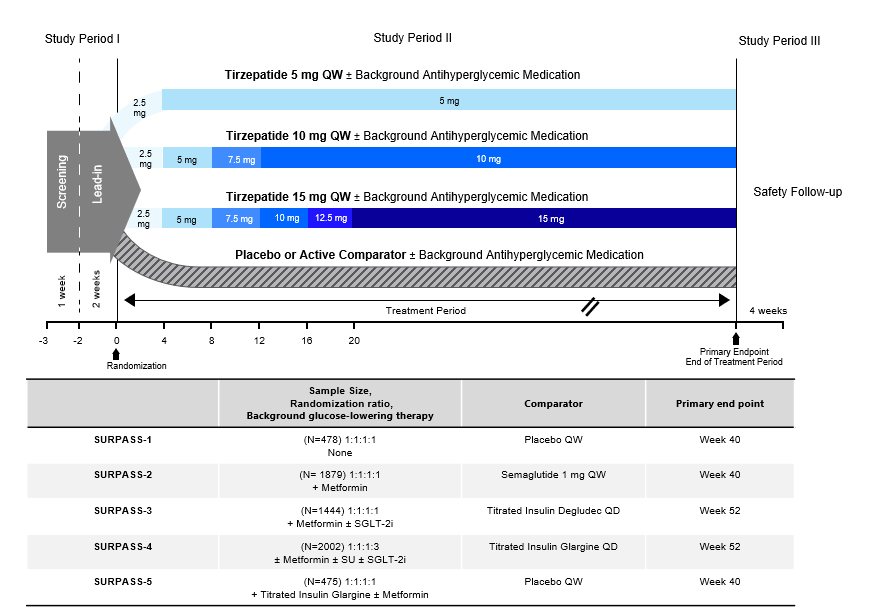


*N* population size; *QD* once daily; *QW* once weekly; *SGLT-2i* sodium-glucose co-transporter 2 inhibitors; *SU* Sulfonylurea
