# Supplementary material for: Systolic blood pressure reduction with tirzepatide in patients with type 2 diabetes: insights from SURPASS clinical program
Source: Cardiovasc Diabetol. 2023 Mar 24;22:66. doi: 10.1186/s12933-023-01797-5 (PMC10039543; doi:10.1186/s12933-023-01797-5)
Supplement: Supplementary file 2 — Additional file 2: Baseline blood pressure across SURPASS studies. [file 12933_2023_1797_MOESM2_ESM.docx]

Additional file 2. Baseline blood pressure across SURPASS studies

Data are mean (SD). *DBP* diastolic blood pressure; *SBP* systolic blood pressure; *TZP* tirzepatide


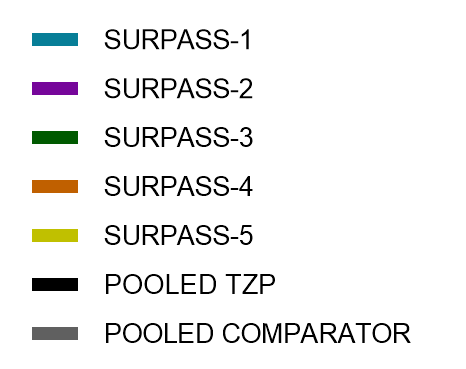


SURPASS-1 (N=478)

SURPASS-2 (N=1878)

SURPASS-3 (N=1437)

SURPASS-4 (N=1995)

SURPASS-5 (N=475)

POOLED COMPARATOR (N=2064)

POOLED TZP (N=4199)
