# Supplementary material for: Systolic blood pressure reduction with tirzepatide in patients with type 2 diabetes: insights from SURPASS clinical program
Source: Cardiovasc Diabetol. 2023 Mar 24;22:66. doi: 10.1186/s12933-023-01797-5 (PMC10039543; doi:10.1186/s12933-023-01797-5)
Supplement: Supplementary file 3 — Additional file 3: Mediation analyses for systolic blood pressure using weight loss as a factor at Week 40/42 (SURPASS 1–5, pooled data for TZP dose). [file 12933_2023_1797_MOESM3_ESM.docx]

**Additional File 3.** Mediation analyses for systolic blood pressure using weight loss as a factor at Week 40/42 (SURPASS 1-5, pooled data for TZP dose)

Data are least-squares mean ETD (95% CI). Data are taken from the safety population of each study. Percentage values represent the percent of blood pressure reduction mediated by weight loss.

*CI* confidence interval; *ETD* estimated treatment difference; *TZP* tirzepatide; *WL-D* weight-loss dependent; *WL-IND* weight-loss independent.
